# Toward therapeutic drug monitoring of citalopram in depression? Insights from a systematic review

**DOI:** 10.3389/fpsyt.2023.1144573

**Published:** 2023-04-27

**Authors:** Na Xu, Zaiwei Song, Dan Jiang, Rongsheng Zhao

**Affiliations:** ^1^Department of Pharmacy, Peking University Third Hospital, Beijing, China; ^2^Department of Pharmacy, Hebei Provincial Mental Health Center, Baoding, China; ^3^Institute for Drug Evaluation, Peking University Health Science Center, Beijing, China; ^4^Therapeutic Drug Monitoring and Clinical Toxicology Center, Peking University, Beijing, China

**Keywords:** citalopram, therapeutic drug monitoring, plasma concentration, depression, treatment outcome

## Abstract

**Background:**

Within the framework of individualized psychopharmacotherapy, therapeutic drug monitoring (TDM) has gained increasing relevance. In the absence of high-quality evidence, the TDM of citalopram (CIT) and the recommended therapeutic ranges of the plasma concentrations have been proposed by guidelines. However, the correlation between the plasma concentration of CIT and treatment outcomes has not been well established. Therefore, the aim of this systematic review was to evaluate the relationship between plasma CIT concentration and treatment outcomes in depression.

**Research design and methods:**

PubMed, Embase, Cochrane Central Register of Controlled Trials (CENTRAL), and Chinese databases (CNKI, Wanfang Data and Sinomed) were searched up to August 6, 2022. We included clinical studies evaluating the correlation between the plasma CIT concentration and treatment outcomes in patients with depression receiving CIT treatment. Outcomes measured included efficacy, safety, medication adherence, and cost-related outcomes. A narrative synthesis was performed to summarize findings from individual studies. This study was performed according to the Preferred Reporting Items for Systematic Reviews, Meta-Analysis (PRISMA) and the reporting guideline for Synthesis without meta-analysis (SWiM).

**Results:**

Eleven studies involving 538 patients were included in total. The reported outcomes were mainly efficacy (*n* = 11) and safety (*n* = 3); one study reported the duration of hospitalization, and no study reported medication adherence. Regarding the efficacy outcomes, three studies revealed the plasma CIT concentration-response relationship and proposed a lower limit of 50 or 53 ng/mL, whereas this was not found in the rest of the studies. Regarding adverse drug events (ADEs), one study reported more ADEs in the low-concentration group (<50 ng/mL vs. >50 ng/mL), which is not convincing from the perspective of pharmacokinetics/pharmacodynamics. Regarding the cost-related outcomes, only one study reported that the high CIT concentration group (≥50 ng/mL) contributed to shortening the hospitalization duration, but it did not provide detailed information, including direct medical expenses and multiple potential factors contributing to longer hospital stays.

**Conclusions:**

A definite correlation between plasma concentration and clinical or cost-related outcomes of CIT cannot be drawn, whereas a tendency toward improved efficacy in patients with plasma concentration above 50 or 53 ng/mL was suggestive from limited evidence.

## 1. Introduction

Depression is a common psychological condition affecting more than 322 million people worldwide (1). Selective serotonin reuptake inhibitors (SSRIs) constitute a large part of antidepressants (2). As one of the representative SSRI drugs, citalopram (CIT) is a widely used and well-tolerated antidepressant in the treatment of depression. However, the response rates in trials have been estimated at only 50–60% (3). Therapeutic drug monitoring (TDM) is the clinical practice of measuring drug exposure at designated intervals to tailor drug doses, thereby optimizing outcomes in individual patients (4). The past years have witnessed great progress in TDM in the field of psychotropic drugs (5).

Within the framework of individualized psychopharmacotherapy, TDM has gained increasing relevance (6). With regard to CIT, polymorphism of CYP2C19 plays an important role in the N-demethylation of CIT *in vivo*. Extensive and poor metabolizers of CYP2C19 caused a significant difference in the behavior of CIT (7). Therefore, TDM has the potential to improve the outcomes of patients receiving CIT therapy. Currently, the TDM of CIT and the definition of therapeutic ranges of the plasma concentrations are recommended as the first level by the TDM expert group of the Arbeitsgemeinschaft für Neuropsychopharmakologie und Pharmakopsychiatrie (AGNP) guideline (2017) (4) as well as a Chinese expert consensus (2022) (8), whereas the TDM of other SSRIs is at the secondary or tertiary level. However, these recommendations were formulated in the absence of high-quality evidence.

The process of TDM is predicated on the assumption that there is a definable relationship between concentration and therapeutic or adverse effects (9), since the dose modifications must rely on the definable relationship. Nevertheless, although TDM is widely used in CIT, the relationship between CIT exposure and treatment outcomes has not been well established in depression. Furthermore, to a certain extent, TDM is costly and time consuming for patients and clinical staff.

Herein, we conducted a systematic review to evaluate the relationship between plasma CIT concentration and treatment outcomes in patients with depression to provide an evidence-based reference for further implementation of TDM in depression.

## 2. Methods

This study was performed according to the Preferred Reporting Items for Systematic Reviews, Meta-Analysis (PRISMA) statement (10) and the reporting guideline for Synthesis without meta-analysis (SWiM) in systematic reviews (11). The PRISMA checklist is included in Supplementary Table S1. The protocol for this systematic review has been registered in the International Prospective Register of Systematic Reviews (PROSPERO, No. CRD42022356425).

### 2.1. Search strategy

Electronic databases, including PubMed, Embase, Cochrane Central Register of Controlled Trials (CENTRAL), and Chinese databases (CNKI, Wanfang Data, and SineMed), were searched for potentially relevant studies from inception to August 6, 2022. Specific search strategies were developed for each database. The combination of keywords (“Citalopram” OR “CIT” OR “Celexa” OR “Lu10171” OR “SSRIs”) AND (“Drug monitoring” OR “Plasma level” OR “TDM” OR “Pharmacokinetics” OR “Drug clearance”) was used to search the title and abstract of the queried literature (Supplementary material II). No restrictions were placed on the study design or language. The search strategy was confirmed by an experienced information library specialist. The reference lists of previous guidelines, expert consensus, reviews (4, 6–8, 12) and included literature were searched for relevant studies.

### 2.2. Eligibility criteria and study selection

Studies were considered eligible if they satisfied all the following inclusion criteria: (1) type of studies: any study evaluating the correlation between the plasma concentration and treatment outcomes; (2) type of subject: patients with depression (including depressed or major depression) received CIT treatment, with no restrictions on ethnicity, sex or age; (3) types of exposure/comparison: measuring the plasma levels of CIT and its metabolites, and determining the correlation between drug exposure and efficacy, safety, adherence or cost-related outcomes; and (4) types of outcomes measured: (i) Efficacy: improvement measured by the Hamilton Depression Scale (HAMD/HDRS), Montgomery-Asberg Depression Rating Scale (MADRS), Clinical Global Impression-Severity (CGI-S), Children's Depression Rating Scale Revised (CDRS-R), and Cronholm-Ottosson Depression Rating Scale (CORS); (ii) Safety: adverse drug events (ADEs) during the CIT treatment, including dry mouth, gastrointestinal reactions, neuropsychiatric side effects, palpitations or QT interval prolongation, etc.; (iii) Medication adherence: the extent to which a patient's behavior corresponds with the prescribed medication dosing regimen; and (iv) Cost-related outcomes: hospitalization length, drug cost, hospitalization cost and other medical expense. Duplicate publications, literatures published in non-English or non-Chinese language, abstracts with not available full texts, unqualified data or unable to extract data were excluded, and only the most recent and comprehensive data were included in the systematic review in the case of overlapping data.

Two authors (X.N. and J.D.) independently assessed the eligibility of all studies based on the inclusion and exclusion criteria above after reviewing the study title, abstract and full text in succession. Studies were included in only the systematic review (but not the meta-analysis) if their findings were relevant to the research question but data were not available for quantitative analysis. Any disagreement among authors was discussed and reconciled by the corresponding author (Z.R.S.).

### 2.3. Data extraction

Two authors (X.N. and S.Z.W.) independently extracted data based on a predesigned standardized extraction form, including the first author, publication year, country, study design, disease, diagnostic criteria, rating scales, age, outcomes, number of patients, therapy duration, clinical efficacy outcome measures, and correlation between efficacy, safety, and plasma CIT levels.

### 2.4. Quality assessment/risk of bias assessment

Two authors (X.N. and S.Z.W.) independently assessed the quality of the included studies. The cohort studies and case-control studies were assessed under the Newcastle-Ottawa Scale (NOS) (13). The NOS attributes a maximum of 9 points to studies based on methodological design and formal reporting, involving “selection”, “comparability” and “exposure/outcome”. NOS scores ranging from 7 to 9 points indicate high quality, 5 to 6 indicate medium quality, and 0 to 4 indicate low quality (14). The Quality In Prognosis Studies (QUIPS) tool (15) was used to assess the quality of the prognosis studies. The QUIPS tool contains six domains, and each domain contains 3–7 prompting items and considerations. Each domain is rated as having a high, moderate, or low risk of bias considering the prompting items. To judge overall risk, the review authors described studies with a low risk of bias as those in which at least 5 of the 6 important bias domains were rated as having a low risk of bias. If there was at least 1 domain rated as high risk or more than 3 domains rated as moderate risk of bias, the overall risk of bias was deemed high. All other variations were determined to have a moderate risk of bias (16). Disagreements regarding quality assessment were resolved by consensus or, when necessary, by consulting the corresponding author.

### 2.5. Statistical analyses

Data were extracted and recorded in Microsoft Excel 2019 by two investigators and subsequently checked by another investigator. Baseline study characteristics were extracted and presented using descriptive statistics. Clinical heterogeneity was estimated by comparing the diagnosis, exposure/comparison, efficacy definition, and other clinical features among studies. Initially, this review was intended as a meta-analysis if valid data assessing the association between CIT plasma concentration and treatment outcomes were available from sufficiently homogeneous studies. However, because of great heterogeneity and a lack of data among different studies, no meta-analysis but a narrative synthesis was performed.

## 3. Results

### 3.1. Electronic searches and study selection

**Figure 1:**
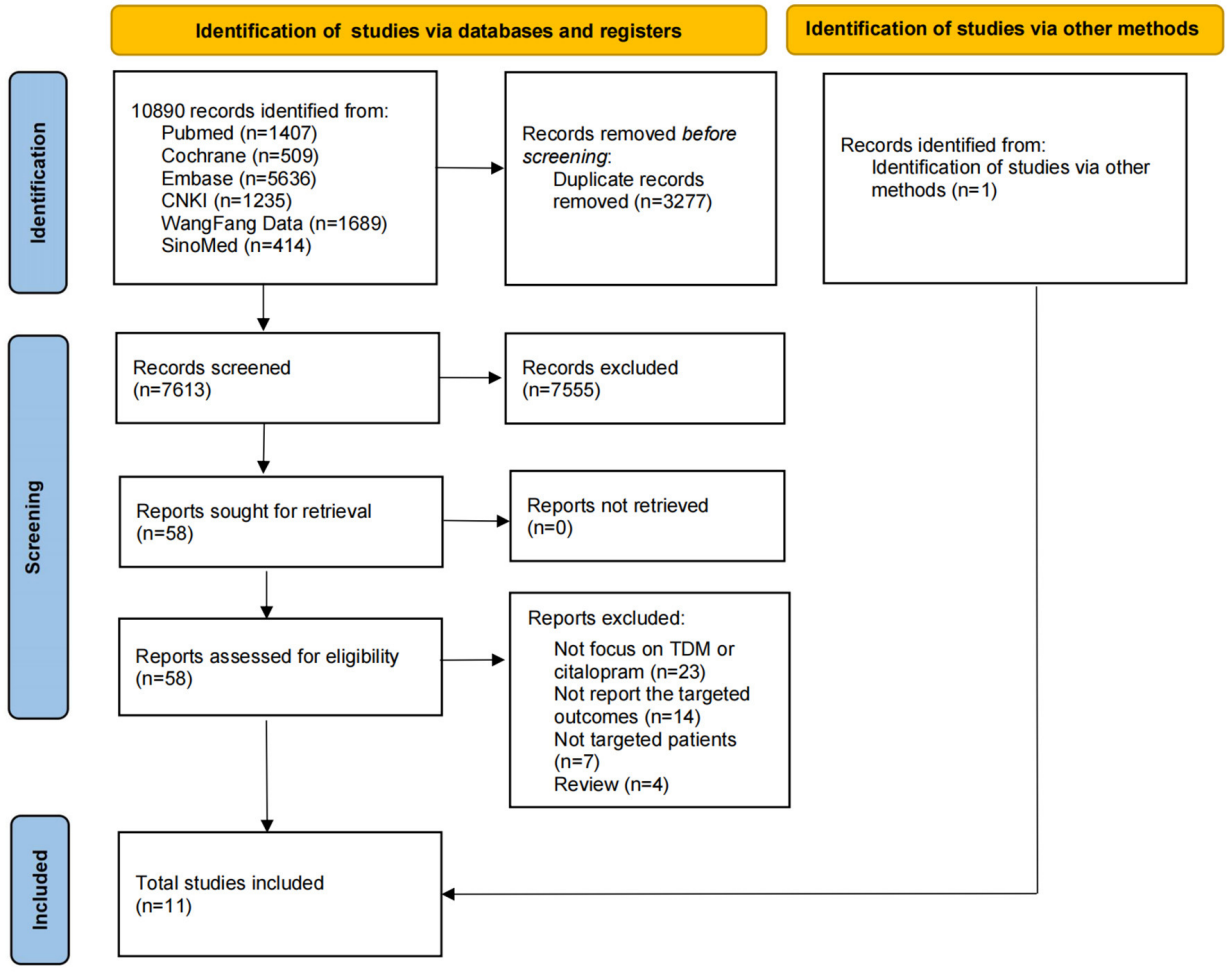
The PRISMA 2020 flow diagram of study selection for the systematic review.

A total of 10,890 candidate references were identified in electronic database searches, and 1 candidate reference was identified using a manual search. After removing duplicate references and carefully reviewing the titles and abstracts, only 58 references were recognized as relevant, and then we assessed all full texts. Of the 58 references, 23 did not focus on TDM or CIT, 14 did not report the targeted outcomes, 7 did not focus on patients with depression, and 4 were reviews. Finally, according to the aforementioned inclusion and exclusion criteria, 11 studies were included in this systematic review. Eleven studies (17–27) were included in the descriptive analysis since the meta-analysis was not feasible. The PRISMA 2020 flow diagram is shown in Figure 1.

### 3.2. Study characteristics and quality assessment

In total, 11 studies involving 538 patients with depression were included. All studies included were published in English. Five studies focused on the correlation between clinical response or non-response and plasma CIT concentration (grouping according to concentration exposure or clinical response), and 6 studies focused on the correlation between score reduction of the rating scales and concentration. In addition, three and one studies focused on the outcomes of ADEs and cost-related outcomes, respectively. In most studies, blood samples were drawn immediately prior to the morning dose of CIT or 8 to 16 h after the night-time dose, when the plasma concentration reached the steady state. The median age of the participants varied from 18 to 88 years. The median follow-up time ranged from 4 to 12 months. The main characteristics of the included studies are summarized in Table 1.

**Table 1:** Main characteristics of the studies included.

**Study ID**	**Country**	**Study design**	**Disease**	**Diagnostic criteria**	**Rating scale**	**Age(y), median (range)**	**Outcome**	**Number of patients (F/M)**	**Therapy duration (weeks)**	**Plasma samples collection**	**Clinical efficacy outcomes measure**	**Correlation of efficacy to CIT Levels (ng/mL)**	**Correlation of Safety to CIT Levels (ng/mL)**
Ozbey et al. (26)	Turkey	Cohort	MDD	DSM-IV	HDRS	37 (18–65)	Efficacy, safety	46(9/37)	6	ss	HDRS	No correlation	**ADE-rate:** C(CIT)_Low − expected_ vs. C(CIT)_High_: *P >* 0.05^b^
Haji et al. (21)^a^	Germany	Cohort	MDD (HAMD-17 ≥ 14)	ICD-10	HAMD-17	49	Efficacy, safety	55(28/27)	5	ss	HAMD	**Clinical response rate:** C(CIT) > 53.0 vs. C(CIT) < 53.0: 53 vs. 17%, *P =* 0.01	**1. Mild-ADE rate:** C(CIT) < 53.0 vs. C(CIT) > 53.0: 44 vs. 22%, the *P* value is not reported. **2. Moderate-ADE rate:** C(CIT) < 53.0 vs. C(CIT) > 53.0: 21 vs. 11%, the *P* value is not reported.
Haji et al. (20)^a^	Germany	Cohort	MDD (HAMD-17 ≥ 14)	ICD-10	HAMD-17	49	Efficacy,Safety, Cost-related outcomes	55(28/27)	5	ss	HAMD	**1. HAMD reduction percentage:** C(CIT)>50.0 vs. C(CIT) < 50.0: *P ≤* 0.019 **2. Mean HAMD**: C(CIT) > 50.0 vs. C(CIT) < 50.0: *P ≤* 0.018 **3. Mean duration hospitalization:** C(CIT) > 50.0 vs. C(CIT) < 50.0: *p* = 0.033	**ADE rate:** C(CIT) < 50.0 vs. C(CIT) > 50.0: *P =* 0.02
Sakolsky et al. (27)	USA	Prognosis	MDD	NR	CGI-S CDRS-R	12–18	Efficacy	27	6	ss	CGI-I CDRS-R	**Clinical response rate:** C(CIT) **≥** GM vs. C(CIT) < GM: 76.5 vs. 30.0%, *P =* 0.04	NR
Nikisch et al. (19)	Germany	Prognosis	MDD (HAMD-21 > 20)	DSM-IV	HAMD-21	19–55	Efficacy	22(13/9)	4	ss	HAMD	No correlation	NR
Amey et al. (24)	Switzerland	Case–control	MDD (HDRS-21 > 18)	DSM-III	HDRS-21	77.1 (67–88)	Efficacy	14(10/4)	4	ss	HDRS	No correlation	NR
Montgomery et al. (23)	UK	Prognosis	MDD (MADRS > 22)	DSM-Ill-R	MADRS	18–70	Efficacy	207(72/135)	12	ss	MADRS	No correlation	NR
Dufour et al. (17)	France	Prognosis	Patients with depression	NR	MADRS	49.3 (29–79)	Efficacy	21(15/6)	4–6	ss	MADRS	No correlation	NR
Bouchard et al. (25)	France	Case–control	Patients with depression (MADRS > 15)	DSM-III	MADRS	44.7 (20–76)	Efficacy	46	6	ss	MADRS	No correlation	NR
Bjerkenstedt et al. (18)	Sweden	Prognosis	Patients with depression	Newcastle Scale II-71	MADRS	25–67	Efficacy	26(19/7)	4	ss	MADRS	No correlation	NR
Pedersen et al. (22)	Denmark	Case–control	Patients with depression (CORS > 5)	Newcastle Scale	CORS	18–60	Efficacy	19	4–6	ss	CORS	No correlation	NR

a The two studies included the same population but reported different outcomes. ss, steady state concentration; MDD, Major depressive disorder; NR, Not reported; ADE, Adverse drug events.

b ADEs included dry mouth, nausea, constipation, palpitation, dizziness, increased perspiration, itching, headache, tremor, blurred vision, difficulty sleeping, sleeping too much, loss of sexual desire, and poor concentration. HAMD/HDRS, Hamilton Depression Scale; CDRS-R, Children's Depression Rating Scale Revised; CGI-S, Clinical Global Impression-Severity; MADRS, Montgomery-Asberg Depression Rating Scale; CORS, Cronholm-Ottosson Depression Rating Scale; GM, geometric mean. Definition of clinical response (dichotomous): (i) a percentage reduction in the initial HAMD/HDRS score ≥50%, (ii) MADRS cutoff scores ≤ 6 points or a percentage reduction in the initial score ≥75%, (iii) CORS cutoff scores ≤ 3 points, (iv) CGI-I score ≤ 2 points, and (v) a percentage reduction in the initial score of CDRS-R ≥ 50%.

Regarding the depression screening instruments, HAMD/HDRS, MADRS, CGI-S, CDRS-R, and CORS were used as instruments for the evaluation of the extent of depression and indicators for the efficacy of the drugs. Seven studies (19–25) reported that patients with depression were scaled as mild-moderate depression by the HAMD, MADRS, and CORS scales. Other studies did not report the patients' scale scores. Regarding the definition of dichotomous clinical response, a percentage reduction of patients' initial HAMD/HDRS score ≥50%, MADRS cutoff scores ≤ 6 points or a percentage reduction of the initial score ≥75%, CGI-I score ≤ 2 points, a percentage reduction in the initial score of CDRS-R ≥ 50%, and CORS cutoff scores ≤ 3 points were defined as clinical response.

The risk of bias assessment is shown in Tables 2, 3. The quality of the cohort and case-control studies evaluated by NOS was 7 or above, indicating a low risk of bias. Five prognosis studies were assessed by QUIPS, three studies were at a low or middle risk of bias, and two studied were considered a high risk of bias since it did not appropriately account for important confounding factors.

**Table 2:** Quality assessment of the cohort and case–control studies.

**Study ID**	**Selection**				**Comparability**	**Exposure/Outcome**			**Score**
	**1**	**2**	**3**	**4**	**5^#^**	**6**	**7**	**8**	
Ozbey et al. (26)^a^	*	*	*		^**^	*	*	*	8
Haji et al. (21)^a^	*	*	*		^**^	*	*	*	8
Haji et al. (20)^a^	*	*	*		*	*	*	*	7
Amey et al. (24)^b^	*	*	*		*	*	*	*	7
Bouchard et al. (25)^b^	*	*	*		^**^	*	*		7
Pedersen et al. (22)^b^	*	*	*		*	*	*	*	7

a Cohort studies: 1. Representativeness of the exposed cohort; 2. Selection of the nonexposed cohort; 3. Ascertainment of exposure; 4. Demonstration that the outcome of interest was not present at the start of the study; 5. Comparability of cohorts on the basis of the design or analysis; 6. Assessment of outcome; 7. Was follow-up long enough for outcomes to occur; 8. Adequacy of follow-up of cohorts.

b Case–control studies: 1. Is the case definition adequate? 2. Representativeness of the cases; 3. Selection of Controls; 4. Definition of Controls; 5. Comparability of cases and controls on the basis of the design or analysis; 6. Ascertainment of exposure; 7. Same method of ascertainment for cases and controls; 8. Nonresponse rate.

# There can be a maximum of two points^*^.

** Indicates that both the most important confounding factors (e.g., age) and other confounding factors were controlled.

**Table 3:** Quality assessment of the prognosis studies.

**Study ID**	**Study participation**	**Study attrition**	**Prognostic factor measurement**	**Outcome measurement**	**Study confounding**	**Statistical analysis and reporting**	**Overall**
Sakolsky et al. (27)	L	H	L	L	M	L	4 L+1 M+1 H
Nikisch et al. (19)	M	L	L	L	M	L	4 L+2 M
Montgomery et al. (23)	L	L	L	L	M	M	4 L+2 M
Dufour et al. (17)	M	M	L	L	L	M	3 L+3 M
Bjerkenstedt et al. (18)	M	M	L	L	H	M	2 L+3 M+1 H

L, Low risk of bias; M, Medium risk of bias; H, High risk of bias.

### 3.3. Clinical efficacy

#### 3.3.1. Clinical response (Dichotomous)

Five studies provided the correlation between clinical response or nonresponse and plasma CIT concentration. Three case-control studies (22, 24, 25) concluded that there was no correlation between plasma CIT concentration and clinical response. One cohort study (21) revealed that, compared to patients with below 53 ng/mL of plasma CIT concentration, patients with above 53 ng/mL (*N* = 19, 35%) showed a significantly higher clinical response rate at day 35 (53 vs. 17%, *P* = 0.01). One prognosis study (27) reported that, compared with patients with lower concentrations, patients with plasma CIT concentrations equal to or greater than the geometric mean value showed a higher rate of response (76.5 vs. 30.0%, *P* = 0.04).

#### 3.3.2. Improvement of scale scores (Continuous)

Six studies provided the relationship between the clinical assessments and plasma CIT concentration. One study (20) revealed that, compared to the low CIT concentration (<50 ng/mL) group, patients above 50 ng/mL showed a better percent reduction in HAMD score (*P* ≤ 0.019) and a lower mean HAMD score (*P* ≤ 0.018). Nevertheless, the other five studies (17–19, 23, 26) concluded that there was no correlation between clinical assessments and plasma CIT concentration.

Regarding the plasma concentration of the major metabolite N-desmethylcitalopram (NDCIT), one study (26) suggested that the high concentration (>73.25 ng/mL) group showed a more significant reduction in HDRS scores than the expected concentration (42.75–73.25 ng/mL) and the low concentration (<42.75 ng/mL) groups (*P* = 0.002). Regarding the concentrations of CIT and NDCIT, the findings are consistent with the above findings (*P* = 0.003).

### 3.4. Clinical safety

Three studies (20, 21, 26) reported the association of plasma CIT concentration and clinical safety, and most ADEs were rated as either mild or moderate. One study (20) revealed that the ADEs rate was significantly higher in the low CIT concentration (<50 ng/mL) group than in the high CIT concentration (>50 ng/mL) group (χ^2^ = 7.7, *P* = 0.02). One study (21) reported that mild or moderate ADEs occurred in 22 and 11%, respectively, in the high group and 44 and 21%, respectively, in the low group, but the detailed *P* value was not provided. Another study (26) showed that dry mouth, nausea, constipation, palpitation, dizziness and other ADEs occurred during CIT therapy, but there was no significant difference between the different concentration groups.

### 3.5. Cost-related outcomes

Only one study (20) reported cost-related outcomes but not direct medical expenses. Patients in the high CIT concentration group (≥50 ng/mL) had a 3-week shorter duration of hospitalization than patients in the low CIT group.

## 4. Discussion

In the field of psychopharmacotherapy, TDM has been reported to not only improve efficacy and safety but also identify medication adherence issues (28, 29). Thus, TDM is considered a valid tool to improve patient outcomes and save healthcare costs in the treatment of depression. The latest clinical guidelines and expert consensus recommended the concentration ranges of CIT at the first recommendation level (4), while we found that the association of TDM-guided CIT concentration-efficacy was not particularly clear in actual clinical practice. Therefore, we paid more attention to the relationship between plasma CIT concentration and treatment outcomes in patients with depression in the present study.

### 4.1. Overall findings and trends

This review revealed four important findings. First, a definite correlation between plasma concentration and clinical efficacy cannot be drawn from inconsistent findings. However, limited studies have supported that the clinical efficacy of patients with plasma CIT concentrations above 50 or 53 ng/mL was better than that of patients with plasma CIT concentrations below 50 or 53 ng/mL. Second, a definite correlation between plasma concentration and clinical safety cannot be drawn in this review. More ADEs appeared to be associated with lower concentrations, but this result is not convincing from the perspective of pharmacokinetics/pharmacodynamics. From the perspective of long-term medication, considering the large utilization of CIT and the evidence gap, CIT-related safety should be paid more attention to (30). Third, with regard to the therapeutic range of CIT (50–110 ng/mL) recommended by clinical guidelines and expert consensus, no evidence was found on the upper limit of concentration. Finally, there is still a lack of evidence on the benefit of CIT TDM in medication adherence and cost savings.

### 4.2. Potential mechanisms and comparison with previous studies

Imaging studies have shown that concentrations of CIT correlate with serotonin transporter (5-HTT) occupancy (31). The study used [^11^C]N,N-Dimethyl-2-(2-amino-4-cyanophenylthio) benzylamine ([^11^C]DASB) positron emission tomography to measure occupancies of SSRIs at minimum therapeutic doses. For SSRIs, as the dose (or plasma level) increased, the occupancy increased non-linearly, with a plateau for higher doses. It was assumed for SSRIs that 80% occupancy of 5-HTT should be attained for maximal clinical improvement. With regard to CIT, this requires serum concentrations of 50 ng/mL or higher (31), which corresponds to the findings in the present review. However, there were no consistent results between the included studies regarding efficacy and concentration. When interpreting results on concentration-efficacy association, heterogeneity between studies needs to be fully considered. Some factors, such as depression status, different scales and cutoff scores, cognitive status, and medication compliance, might have implications for the interpretation of the overall results.

It has been reported that neuropsychopharmacological drugs tend to exhibit two opposite directions of effect across their respective ranges of concentrations. In lower concentration ranges, there seems to be a positive direction of effect, increasing efficacy with increasing concentrations. In contrast, in higher concentration ranges, a negative direction could be observed, indicating a decline in efficacy with increasing concentrations (12). However, we did not find any data on the upper cut-off value of CIT concentrations in this study, although the recommended therapeutic reference range of CIT is 50–110 ng/mL by AGNP (4).

CIT is predominantly eliminated by cytochrome P450 (CYP)-catalyzed oxidation in the liver. CIT is partially N-demethylated to desmethylcitalopram (DCIT) by CYP2C19 and CYP3A4, and DCIT is further N-demethylated to NDCIT by CYP2D6 (32). In our review, the plasma levels of DCIT or NDCIT were also measured in six studies (17, 19, 22–24, 26), but only one study showed that the concentrations of NDCIT or CIT and NDCIT may be associated with the improvement in HDRS scores. According to previous consensus, the concentrations of DCIT and NDCIT are 30–50% and 5–10%, respectively, and the two metabolites are not active and are thought not to contribute to antidepressant activity (32).

Although TDM has been considered to identify medication adherence issues in psychopharmacotherapy, the role of conducting TDM in CIT medication adherence was not reported in any study included. Regarding the cost-related outcomes, only one indirect study (20) revealed that high concentrations (≥50 ng/mL) of CIT would help to shorten the hospitalization duration, but it did not provide other details or direct cost data. Since there are multiple potential factors contributing to longer hospital stays (e.g., patient insurance, hospital policy, funding, etc.), the results of this study need to be interpreted with caution. Additionally, one previous study reported that the TDM of SSRIs has the potential to reduce drug costs in elderly patients with depression (33). Based on the above considerations, higher quality studies are needed to validate the economic benefit of CIT TDM.

As the associations between concentration and clinical effects of antidepressants have become a wide clinical concern, some reviews have been published earlier. One systematic review published in 2022 (12) focused on the association between the concentration and clinical effect of antidepressants, which were divided into several categories such as SSRI, tricyclic antidepressants (TCA), tetracyclic antidepressants (Tetra-CA), and selective serotonin-noradrenaline reuptake-inhibitors (SSNRI), but did not provide detailed information on CIT or escitalopram to guide clinical practice. From the perspective of review findings, both the previous review and our present review reported that research on the association between the concentration and clinical effect of antidepressants has yielded ambiguous results. Another systematic review published in 2020 (6) discussed a concentration-effect relationship for 11 psychotropic drugs in children and adolescents and found that the evidence is sparse and therapeutic reference ranges are generally not evaluated or reported.

### 4.3. Limitations

Several limitations should be considered in our review. First, the data were derived from studies with different study designs, efficacy assessments and outcome definitions. The substantial heterogeneity among the studies remained largely unexplained and thus may contribute to discrepancies in evaluation results. In addition, methodological shortcomings in primary studies might systematically influence the relationship between the variables. Second, the analysis relied on a limited number of original studies, and the sample size included in these selected studies was insufficient. Third, due to the substantial heterogeneity or the limited number of studies, meta-analyses could not be performed to draw more definitive conclusions. The aforementioned limitations warrant future larger validation studies of the association between plasma concentrations and treatment outcomes of CIT in patients with depression.

### 4.4. Recommendation for clinical practice

In light of the findings in this study, the association of CIT plasma concentration and treatment outcomes in patients with depression remains inconclusive. From a clinician or pharmacist's point of view, we propose some suggestions on the clinical implementation of CIT TDM. First, for patients with satisfactory response and stable condition after CIT treatment, it is not recommended to carry out TDM routinely. In the case of insufficient clinical improvement, CIT TDM could be carried out, and the dose should be optimized to achieve a concentration above 50 or 53 ng/mL. Usually, the corresponding dose range to achieve this goal is 20 to 40 mg per day with a median level of 30 mg (21). Second, TDM may be useful under some certain conditions, such as patients with pregnancy, elderly patients, patients with liver impairment, and patients taking other medications that may interact with CIT. Third, given the lack of evidence on the upper limit of TDM from a safety perspective, concentration monitoring might offer some information on the ADE etiology only in the presence of ADEs. Last but not least, we would like to encourage clinicians or pharmacists to accumulate real-world evidence of the clinical or economic benefit of TDM in their clinical practice.

## 5. Conclusion

In summary, a definite correlation between plasma concentration and clinical or cost-related outcomes cannot be drawn from current findings, whereas a tendency toward improved efficacy in patients with plasma CIT concentrations above 50 or 53 ng/mL was suggestive. Therefore, we recommend that TDM for CIT be considered under certain conditions, such as patients who are pregnant, elderly patients, patients with liver impairment, patients taking other medications that may interact with CIT, and patients with insufficient clinical improvement.

## Data availability statement

The original contributions presented in the study are included in the article/Supplementary material, further inquiries can be directed to the corresponding author.

## Author contributions

RZ and ZS conceived and designed the study. NX and ZS collected and analyzed the data and performed the statistical analysis and wrote the article. NX and DJ prepared the pictures and tables. RZ provided suggestions and participated in the revision of the article. All authors read and approved the final manuscript.
