# Supplementary material for: Toward therapeutic drug monitoring of citalopram in depression? Insights from a systematic review
Source: Front Psychiatry. 2023 Apr 27;14:1144573. doi: 10.3389/fpsyt.2023.1144573 (PMC10174233; doi:10.3389/fpsyt.2023.1144573)
Supplement: Supplementary file 1 [file Data_Sheet_1.docx]

**Supplemental Materials**

Content

[Supplemental Material I 2](#_Toc18337)

[Table S1. PRISMA Checklist 2](#_Toc28158)

[Supplemental Material II – Search strategies 5](#_Toc31672)

[PubMed 5](#_Toc9479)

[Embase 5](#_Toc29391)

[Cochrane Central Register of Controlled Trials (CENTRAL) 6](#_Toc10442)

[SinoMed 6](#_Toc29391)

[CNKI 7](#_Toc29391)

[Wanfang Data 7](#_Toc29391)

[Supplemental Material Ⅲ– Excluded studies list 8](#_Toc31672)

## Supplemental Material I

### Table S1. PRISMA Checklist

| **Section/Topic** | **Item** | **Checklist item** | **Page** |
| --- | --- | --- | --- |
| **Title** | | |  |
| Title | 1 | Identify the report as a systematic review, meta-analysis, or both | P1 |
| **ABSTRACT** | | |  |
| Structured summary | 2 | Provide a structured summary including, as applicable, background, objectives, data sources, study eligibility criteria, participants, interventions, study appraisal and synthesis methods, results, limitations, conclusions and implications of key findings, systematic review registration number | P2 |
| **INTRODUCTION** | | |  |
| Rationale | 3 | Describe the rationale for the review in the context of what is already known | P3 |
| Objectives | 4 | Provide an explicit statement of questions being addressed with reference to participants, interventions, comparisons, outcomes, and study design (PICOS) | P4 |
| **METHODS** | | |  |
| Protocol and registration | 5 | Indicate if a review protocol exists, if and where it can be accessed (such as web address), and, if available, provide registration information including registration number | P3 |
| Eligibility criteria | 6 | Specify study characteristics (such as PICOS, length of follow-up) and report characteristics (such as years considered, language, publication status) used as criteria for eligibility, giving rationale | P3-4 |
| Information sources | 7 | Describe all information sources (such as databases with dates of coverage, contact with study authors to identify additional studies) in the search and date last searched | P4 |
| Search | 8 | Present full electronic search strategy for at least one database, including any limits used, such that it could be repeated | Supple II |
| Study selection | 9 | State the process for selecting studies (that is, screening, eligibility, included in systematic review, and, if applicable, included in the meta-analysis) | P4 |
| Data collection process | 10 | Describe method of data extraction from reports (such as piloted forms, independently, in duplicate) and any processes for obtaining and confirming data from investigators | P4-5 |
| Data items | 11 | List and define all variables for which data were sought (such as PICOS, funding sources) and any assumptions and simplifications made | P4-5 |
| Risk of bias in individual studies | 12 | Describe methods used for assessing risk of bias of individual studies (including specification of whether this was done at the study or outcome level), and how this information is to be used in any data synthesis | P5 |
| Summary measures | 13 | State the principal summary measures (such as risk ratio, difference in means) | P5 |
| Synthesis of results | 14 | Describe the methods of handling data and combining results of studies, if done, including measures of consistency (such as I^2^ statistic) for each meta-analysis | P5 |
| Risk of bias across studies | 15 | Specify any assessment of risk of bias that may affect the cumulative evidence (such as publication bias, selective reporting within studies) | P5 |
| Additional analyses | 16 | Describe methods of additional analyses (such as sensitivity or subgroup analyses, meta-regression), if done, indicating which were pre-specified | P5 |
| **RESULTS** | | |  |
| Study selection | 17 | Give numbers of studies screened, assessed for eligibility, and included in the review, with reasons for exclusions at each stage, ideally with a flow diagram | P5-6 |
| Study characteristics | 18 | For each study, present characteristics for which data were extracted (such as study size, PICOS, follow-up period) and provide the citations | P6 |
| Risk of bias within studies | 19 | Present data on risk of bias of each study and, if available, any outcome-level assessment (see item 12). | P6 |
| Results of individual studies | 20 | For all outcomes considered (benefits or harms), present for each study (a) simple summary data for each intervention group and (b) effect estimates and confidence intervals, ideally with a forest plot | P6-7 |
| Synthesis of results | 21 | Present results of each meta-analysis done, including confidence intervals and measures of consistency | P6-7 |
| Risk of bias across studies | 22 | Present results of any assessment of risk of bias across studies (see item 15) | P7 |
| Additional analysis | 23 | Give results of additional analyses, if done (such as sensitivity or subgroup analyses, meta-regression) (see item 16) | P7 |
| **DISCUSSION** | | |  |
| Summary of evidence | 24 | Summarise the main findings including the strength of evidence for each main outcome; consider their relevance to key groups (such as health care providers, users, and policy makers) | P7-10 |
| Limitations | 25 | Discuss limitations at study and outcome level (such as risk of bias), and at review level (such as incomplete retrieval of identified research, reporting bias) | P10 |
| Conclusions | 26 | Provide a general interpretation of the results in the context of other evidence, and implications for future research | P10-11 |
| **Funding** | | |  |
| Funding | 27 | Describe sources of funding for the systematic review and other support (such as supply of data) and role of funders for the systematic review | P11 |

## Supplemental Material II – Search strategies

### PubMed

**#1** Citalopram[MeSH] OR Citalopram[Title/Abstract] OR CIT[Title/Abstract] OR Cipramil[Title/Abstract] OR Celexa[Title/Abstract] OR Lu10171[Title/Abstract] OR Lu-10-171[Title/Abstract] OR Citalopram Hydrobromide[Title/Abstract] OR Cytalopram[Title/Abstract] OR Seropram[Title/Abstract]

**#2** "Selective serotonin re-uptake inhibitors"[Title/Abstract] OR "Selective serotonin reuptake inhibitors"[Title/Abstract] OR "SSRI"[Title/Abstract] OR "SSRIs"[Title/Abstract]

**#3** ("Drug Monitoring"[Title/Abstract] OR "Pharmacokinetics"[Title/Abstract] OR "Drug Clearance"[Title/Abstract] OR "Plasma Clearance"[Title/Abstract] OR "Clearance"[Title/Abstract] OR "Metabolic Clearance Rate"[Title/Abstract] OR "Metabolic Clearance"[Title/Abstract] OR "Blood Level"[Title/Abstract] OR "Concentration at Steady-State"[Title/Abstract] OR "Concentration Ratio"[Title/Abstract] OR "Concentration Response"[Title/Abstract] OR "Drug Blood Level"[Title/Abstract] OR "Drug Concentration"[Title/Abstract] OR "Maximum Concentration"[Title/Abstract] OR "Maximum Plasma Concentration"[Title/Abstract] OR "Minimum Concentration"[Title/Abstract] OR "Minimum Effective Concentration"[Title/Abstract] OR "Minimum Plasma Concentration"[Title/Abstract] OR "Plasma Concentration-Time Curve"[Title/Abstract] OR "Time to Maximum Plasma Concentration"[Title/Abstract] OR "TDM"[Title/Abstract] OR "Drug Monitoring"[Title/Abstract] OR "Medication Monitoring"[Title/Abstract] OR "clearance*"[Title/Abstract] OR "pharmacokinetic*"[Title/Abstract] OR "Concentration"[Title/Abstract] OR "CMax"[Title/Abstract] OR "CMin"[Title/Abstract] OR "TMax"[Title/Abstract] OR "Blood Level"[Title/Abstract] OR "Drug Level"[Title/Abstract] OR "Plasma Level"[Title/Abstract] OR "Serum Level"[Title/Abstract])

**#4 (#1 OR #2) AND #3**

### Embase

**#1** 'Citalopram'/exp OR 'Citalopram':ab,ti OR 'CIT':ab,ti OR 'Cipramil':ab,ti OR 'Celexa':ab,ti OR 'Lu10171':ab,ti OR 'Lu-10-171':ab,ti OR 'Citalopram Hydrobromide':ab,ti OR 'Cytalopram':ab,ti OR 'Seropram':ab,ti

**#2** 'Selective serotonin re-uptake inhibitors'/exp OR 'Selective serotonin re-uptake inhibitors':ab,ti OR 'Selective serotonin reuptake inhibitors'/exp OR 'Selective serotonin reuptake inhibitors':ab,ti OR 'SSRI':ab,ti OR 'SSRIs':ab,ti

**#3** 'Drug Monitoring':ab,ti OR 'Pharmacokinetics':ab,ti OR 'Drug Clearance':ab,ti OR 'Drug Clearance Ratio':ab,ti OR 'Plasma Clearance':ab,ti OR 'Clearance':ab,ti OR 'Metabolic Clearance Rate':ab,ti OR 'Metabolic Clearance':ab,ti OR 'Blood Level':ab,ti OR 'Concentration at Steady-State':ab,ti OR 'Concentration Ratio':ab,ti OR 'Concentration Response':ab,ti OR 'Drug Blood Level':ab,ti OR 'Drug Concentration':ab,ti OR 'Exp Effective Concentration':ab,ti OR 'Maximum Concentration':ab,ti OR 'Maximum Plasma Concentration':ab,ti OR 'Minimum Concentration':ab,ti OR 'Minimum Effective Concentration':ab,ti OR 'Minimum Plasma Concentration':ab,ti OR 'Plasma Concentration-Time Curve':ab,ti OR 'Time to Maximum Plasma Concentration':ab,ti OR 'Clearance at Steady-State':ab,ti OR ('TDM':ab,ti OR 'Drug Monitoring':ab,ti OR 'Medication Monitoring':ab,ti OR 'Clearance*':ab,ti OR 'Pharmacokinetic*':ab,ti OR 'Concentration*':ab,ti OR 'CMax':ab,ti OR 'CMin':ab,ti OR 'TMax':ab,ti OR 'Blood Level':ab,ti OR 'Drug Level':ab,ti OR 'Plasma Level':ab,ti OR 'Serum Level':ti,ab)

**#4 (#1 OR #2) AND #3**

### Cochrane Central Register of Controlled Trials (CENTRAL)

**#1** Citalopram[MeSH] OR Citalopram:ti,ab,kw OR CIT:ti,ab,kw OR Cipramil:ti,ab,kw OR 'Celexa':ti,ab,kw OR 'Lu10171':ti,ab,kw OR 'Citalopram Hydrobromide':ti,ab,kw OR 'Cytalopram':ti,ab,kw OR 'Seropram':ti,ab,kw

**#2** 'Selective serotonin re-uptake inhibitors':ti,ab,kw OR 'Selective serotonin reuptake inhibitors':ti,ab,kw OR 'SSRI':ti,ab,kw OR 'SSRIs':ti,ab,kw

**#3** ('Drug Monitoring' OR 'Pharmacokinetics' OR 'Drug Clearance' OR 'Drug Clearance Ratio' OR 'Plasma Clearance' OR 'Clearance' OR 'Metabolic Clearance Rate' OR 'Metabolic Clearance' OR 'Blood Level' OR 'Concentration at Steady-State' OR 'Concentration Ratio' OR 'Concentration Response' OR 'Drug Blood Level' OR 'Drug Concentration' OR 'Exp Effective Concentration' OR 'Maximum Concentration' OR 'Maximum Plasma Concentration' OR 'Minimum Concentration' OR 'Minimum Effective Concentration' OR 'Minimum Plasma Concentration' OR 'Plasma Concentration-Time Curve' OR 'Time to Maximum Plasma Concentration' OR 'Clearance at Steady-State'):ti,ab,kw OR ('TDM' OR 'Drug Monitoring' OR 'Medication Monitoring' OR 'Clearance*' OR 'TMax' OR 'Blood Level' OR 'Drug Level' OR 'Plasma Level' OR vSerum Level'):ti,ab,kw

**#4 (#1 OR #2) AND #3**

### SinoMed

**#1** “西酞普兰”[标题:智能] OR “西酞普兰”[摘要:智能] OR “喜普妙”[标题:智能] OR “喜普妙”[摘要:智能]

**#2** “新型抗抑郁药”[标题:智能]“新型抗抑郁药”[摘要:智能] OR “选择性5羟色胺再摄取抑制剂”[标题:智能] OR “选择性5羟色胺再摄取抑制剂”[摘要:智能] OR “选择性5-羟色胺(5-HT)再摄取抑制剂”[标题:智能] OR “选择性5-羟色胺(5-HT)再摄取抑制剂”[摘要:智能] OR “选择性5-羟色胺再摄取抑制剂”[标题:智能] OR “选择性5-羟色胺再摄取抑制剂”[摘要:智能] OR “5-羟色胺再摄取抑制剂”[标题:智能] OR “5-羟色胺再摄取抑制剂”[摘要:智能]

**#3** “药物监测”[标题:智能] OR “药物监测”[摘要:智能] OR “浓度”[标题:智能] OR “浓度” [摘要:智能] OR “水平”[标题:智能] OR “水平”[摘要:智能] OR “药代动力学”[标题:智能] OR “药代动力学”[摘要:智能] OR “药物动力学”[标题:智能] OR “药物动力学”[摘要:智能] OR “药动学”[标题:智能] OR “药动学”[摘要:智能] OR “稳态”[标题:智能] OR “稳态”[摘要:智能] OR “代谢”[标题:智能] OR “代谢”[摘要:智能] OR “排泄”[标题:智能] OR “排泄”[摘要:智能] OR “清除”[标题:智能] OR “清除”[摘要:智能] OR “消除”[标题:智能] OR “消除” [摘要:智能] OR “TDM”[标题:智能] OR “TDM”[摘要:智能]

**#4 (#1 OR #2) AND #3**

### CNKI

**#1** TI=‘西酞普兰’ OR AB=‘西酞普兰’ OR TI=‘喜普妙’ OR AB=‘喜普妙’

**#2** TI=‘新型抗抑郁药’ OR AB=‘新型抗抑郁药’ OR TI=‘选择性5-羟色胺(5-HT)再摄取抑制剂’ OR AB=‘选择性5-羟色胺(5-HT)再摄取抑制剂’ OR TI=‘选择性5-羟色胺再摄取抑制剂’ OR AB=‘选择性5-羟色胺再摄取抑制剂’ OR TI=‘5-羟色胺再摄取抑制剂’ OR AB=‘5-羟色胺再摄取抑制剂’

**#3** TI=‘药物监测’ OR AB=‘药物监测’ OR TI=‘浓度’ OR AB=‘浓度’ OR TI=‘水平’ OR AB=‘水平’ OR TI=‘药代动力学’ OR AB=‘药代动力学’ OR TI=‘药物动力学’ OR AB=‘药物动力学’ OR TI=‘药动学’ OR AB=‘药动学’ OR TI=‘稳态’ OR AB=‘稳态’ OR TI=‘代谢’ OR AB=‘代谢’ OR TI=‘排泄’ OR AB=‘排泄’ OR TI=‘清除’ OR AB=‘清除’ OR TI=‘消除’ OR AB=‘消除’ OR TI=‘TDM’ OR AB=‘TDM’

**#4 (#1 OR #2) AND #3**

### Wanfang Data

**#1** 题名：(西酞普兰) OR 摘要：(西酞普兰) OR 题名：(喜普妙) OR 摘要：(喜普妙)

**#2** 题名：(新型抗抑郁药) OR 摘要：(新型抗抑郁药) OR 题名：(选择性5-羟色胺(5-HT)再摄取抑制剂) OR 摘要：(选择性5-羟色胺(5-HT)再摄取抑制剂) OR 题名：(选择性5-羟色胺再摄取抑制剂) OR 摘要：(选择性5-羟色胺再摄取抑制剂) OR 题名：(5-羟色胺再摄取抑制剂) OR 摘要：(5-羟色胺再摄取抑制剂)

**#3**题名：(药物监测) OR 摘要：(药物监测) OR 题名：(浓度) OR 摘要：(浓度) OR 题名：(水平) OR 摘要：(水平) OR 题名：(药代动力学) OR 摘要：(药代动力学) OR 题名：(药动学) OR 摘要：(药动学) OR 题名：(稳态) OR 摘要：(稳态) OR 题名：(排泄) OR 摘要：(排泄) OR 题名：(清除) OR 摘要：(清除) OR 题名：(消除) OR 摘要：(消除) OR 题名：(代谢) OR摘要：(代谢) OR 题名：(TDM) OR 摘要：(TDM)

**#4 (#1 OR #2) AND #3**

### Supplemental Material Ⅲ

### Excluded studies list

**1. Studies not focus on TDM or CIT**

[1] Viscogliosi G, Chiriac IM, Ettorre E. Efficacy and Safety of Citalopram Compared to Atypical Antipsychotics on Agitation in Nursing Home Residents With Alzheimer Dementia. J Am Med Dir Assoc. 2017;18(9):799-802.

[2] Parmar S, Kataria D. A comparative study of hyponatraemia caused by the use of different types of selective serotonin re-uptake inhibitors. Indian Journal of Psychiatry 2012;54:76.

[3] Milne RJ, Goa KL. Citalopram-A review of its pharmacodynamic and pharmacokinetic properties, and therapeutic potential in depressive illness. Drugs. 1991;41(3):450-477.

[4] Herrmann N, Black SE, Chow T, Cappell J, Tang-Wai DF, Lanctôt KL. Serotonergic function and treatment of behavioral and psychological symptoms of frontotemporal dementia. Am J Geriatr Psychiatry. 2012;20(9):789-797.

[5] Gutierrez M, Abramowitz W. Steady-state pharmacokinetics of citalopram in young and elderly subjects. Pharmacotherapy. 2000;20(12):1441-1447.

[6] Deniz S, Sancar M, Okuyan B, Ata P. et al. Determination of CYP2≥19 polymorphisms, adverse drug reaction, and medication adherence in patients utilized selective serotonin reuptake inhibitors. 2015;37(1):231-232.

[7] Bschor T, Ising M, Erbe S. et al. Impact of citalopram on the HPA system. A study of the combined DEX/CRH test in 30 unipolar depressed patients. J Psychiatr Res. 2012;46(1):111-117.

[8] Athreya A. P, Neavin D, Frye M, Skime, M. el al. Factor graphs identify sex-specific antidepressant response profiles: Citalopram/escitalopram as molecular probes for subgroups of major depressive disorder patients. 2018;103:9-10.

[9] Apter A, Kroneberg S, Frisch A, Weizmarm A, Wasserman D. The pharmacogenetics of suicidal adverse reactions to SSRI medications in children and adolescents .International Journal of Neuropsychopharmacology.2012;15 Suppl 1:11.

[10] Cipriani A, Koesters M, Furukawa TA, et al. Duloxetine versus other anti-depressive agents for depression. Cochrane Database Syst Rev. 2012;10:CD006533. Published 2012 Oct 17.

[11] Corruble E, Guelfi JD. Is there a relationship between clinical efficacy and antidepressant dosage in major depression?. Encephale. 1999;25 Spec No 2:39-46.

[12] Fabbri C, Marsano A, Balestri M, De Ronchi D, Serretti A. Clinical features and drug induced side effects in early versus late antidepressant responders. J Psychiatr Res. 2013;47(10):1309-1318.

[13] Frommeyer G, Brücher B. et al. Low proarrhythmic potential of citalopram and escitalopram in contrast to haloperidol in an experimental whole-heart model. Eur J Pharmacol. 2016;788:192-199.

[14] Hertz L, Rothman DL, Li B, Peng L. Chronic SSRI stimulation of astrocytic 5-HT2B receptors change multiple gene expressions/editings and metabolism of glutamate, glucose and glycogen: a potential paradigm shift. Front Behav Neurosci. 2015;9:25. Published 2015 Feb 20.

[15] Gram LF. Therapeutic Drug Monitoring in Psychiatry. CNS Drugs. 1995;4(6), 454–455.

[16] Pasupula S, Yedlapalli M, Pasupula R, Meghana S, Kota SK. Efficacy of escitalopram versus desvenlafaxine in treatment of depression: A comparative study from a rural tertiary care hospital. International Journal of Pharmaceutical Research. 2021;13(1):1073-1077.

[17] Power BM, Hackett LP, Dusci LJ, Ilett KF. Antidepressant toxicity and the need for identification and concentration monitoring in overdose. Clin Pharmacokinet. 1995;29(3):154-171.

[18] Prisco V, Iannaccone T, Tusciano A, et al. Drug safety warnings in psychiatry: adverse drug reactions' signaling from 2002 to 2014. Riv Psichiatr. 2016;51(3):96-103.

[19] Ruhe E, Booij J, Van Weert H, Reitsma J, Franssen E, Michel M, Schene A. Increasing SSRIs dosage in depression is not significant. Huisarts en wetenschap. 2009;52(6):289-296.

[20] Schwarz MJ, Hiemke C, Baumann P; AGNP TDM expert group. Clinical relevance of TDM of SSRIs. Ther Drug Monit. 2006;28(5):716-717.

[21] Schwasinger-Schmidt T. E, Macaluso M. Other Antidepressants. Clin Pharmacokinet. 2019;250:325-355.

[22] Sechter D, Lane R. Continuation therapy with selective serotin re-uptake inhibitors. Journal of Serotonin Research. 1997;4(2):65-111.

[23] Vitali M, Tedeschini E, Mistretta M, et al. Adjunctive pregabalin in partial responders with major depressive disorder and residual anxiety. J Clin Psychopharmacol. 2013;33(1):95-98.

**2. Studies with no outcomes**

[1] Baumann P. Pharmacology and pharmacokinetics of citalopram and other SSRIs. Int Clin Psychopharmacol. 1996;11 Suppl 1:5-11.

[2] Baumann P, Nil R, Souche A, et al. A double-blind, placebo-controlled study of citalopram with and without lithium in the treatment of therapy-resistant depressive patients: a clinical, pharmacokinetic, and pharmacogenetic investigation. J Clin Psychopharmacol. 1996;16(4):307-314.

[3] Bjerkenstedt L, Edman G, Flyckt L, Hagenfeldt L, Sedvall G, Wiesel FA. Clinical and biochemical effects of citalopram, a selective 5-HT reuptake inhibitor--a dose-response study in depressed patients. Psychopharmacology (Berl). 1985;87(3):253-259.

[4] Foglia JP, Pollock BG, Kirshner MA, Rosen J, Sweet R, Mulsant B. Plasma levels of citalopram enantiomers and metabolites in elderly patients. Psychopharmacol Bull. 1997;33(1):109-112.

[5] Kumar Y, Kung S, Shinozaki G. CYP2C19 variation, not citalopram dose nor serum level, is associated with QTc prolongation. J Psychopharmacol. 2014;28(12):1143-1148.

[6] Reis M, Chermá MD, Carlsson B, Bengtsson F. Task Force for TDM of Escitalopram in Sweden. Therapeutic drug monitoring of escitalopram in an outpatient setting. Ther Drug Monit. 2007;29(6):758-766.

[7] Reis M, Olsson G, Carlsson B, et al. Serum levels of citalopram and its main metabolites in adolescent patients treated in a naturalistic clinical setting. J Clin Psychopharmacol. 2002;22(4):406-413.

[8] Unterecker S, Pfuhlmann B. Effects of sex and age on serum concentrations of antidepressants under naturalistic conditions. Pharmacopsychiatry. 2011; 21(6).

[9] Unterecker S, Riederer P, Proft F, Maloney J, Deckert J, Pfuhlmann B. Effects of gender and age on serum concentrations of antidepressants under naturalistic conditions. J Neural Transm (Vienna). 2013;120(8):1237-1246.

[10] Valuck RJ, Libby AM, Anderson HO, Erder MH, Francois C ,Doshi JA, Collins C, Preskorn SH. Rates of co-prescribing of drugs with potential for drug-drug interactions among persons initiating therapy with selective serotonin reuptake inhibitors. Journal of General Internal Medicine. 2011;26 Suppl 1:268-268.

[11] Westin AA, Brekke M, Molden E, Skogvoll E, Spigset O. Selective serotonin reuptake inhibitors and venlafaxine in pregnancy: Changes in drug disposition [published correction appears in PLoS One. 2018 Jan 16;13(1):e0191508]. PLoS One. 2017;12(7):e0181082. Published 2017 Jul 14.

[12] Haji EO, Tadic A, Dragicevic A, Müller MJ, Grasmäder K, Rao ML, Laux G, Hiemke C. Plasma concentrations of citalopram in depressed inpatients and clinical improvement in a naturalistic setting. European Archives of Psychiatry and Clinical Neuroscience. 2010; 260: 72.

[13] Darke S, Torok M, Duflou J. Contributory and incidental blood concentrations in deaths involving citalopram. J Forensic Sci. 2013;58(2):432-435. doi:10.1111/1556-4029.12046.

[14] Abanades S,Van der Aart J ,Barletta J,Marzano C,Searle G,Ahmad J,Zamuner S,Cunningham V,Rabiner E ,Laruelle M. Characterising the relationship between plasma pharmacokinetics and occupancy following single dose allows prediction of repeat dose occupancy. Journal of Cerebral Blood Flow and Metabolism.2009;29:64-65.

**3. Studies not focus on depressed patients**

[1] Flores G, Perez-Patrigeon S, Cobos-Ayala C, Vergara J. Severe symptomatic hyponatremia during citalopram therapy-a case report. BMC Nephrol. 2004;5:2. Published 2004 Jan 16.

[2] Henning J, Netter P. Oral application of citalopram (20 mg) and its usefulness for neuroendocrine challenge tests. Int J Neuropsychopharmacol. 2002;5(1):67-71.

[3] Howland RH. A critical evaluation of the cardiac toxicity of citalopram: part 2. J Psychosoc Nurs Ment Health Serv. 2011;49(12):13-16.

[4] Lader M, Melhuish A, Frcka G, Fredricson Overø K, Christensen V. The effects of citalopram in single and repeated doses and with alcohol on physiological and psychological measures in healthy subjects. Eur J Clin Pharmacol. 1986;31(2):183-190.

[5] Linnet K, Olesen OV. Citalopram and desmethylcitalopram for psychiatric patients. Ugeskr Laeger. 1996;158(35):4920-4923.

[6] Klampfl K, Mehler-Wex C, Warnke A, Gerlach M. Therapeutic drug monitoring in child and adolescent psychiatry.Psychopharmakotherapie. 2010;17(4):193-200.

[7] Wichers RH, Findon JL, Jelsma A, et al. Modulation of brain activation during executive functioning in autism with citalopram. Transl Psychiatry. 2019;9(1):286.

**4. Reviews**

[1] Baumann P. Pharmacokinetic-pharmacodynamic relationship of the selective serotonin reuptake inhibitors. Clin Pharmacokinet. 1996;31(6):444-469.

[2] Ostad Haji E,Tadic A, Dragicevic A,Muller MJ, Grasmader K,Rao ML, Laux G, Hiemke C. Plasma concentrations of citalopram in inpatients with acute major depression are predictive for later non-response. Data from an observational study. Pharmacopsychiatry. 2012;44(6).

[3] Paluch Z,Richter T, Sadilkova L, Vyhlidalova I, Alusik S. Antidepressant therapy and TDM. 2017;35(6):1329.

[4] Saglam E. Personalized medicine in psychiatry. Klinik Psikofarmakoloji Bulteni. 2013;23:38-39.
